# Augmenting an online self-directed intervention for gambling disorder with a single motivational interview: study protocol for a randomized controlled trial

**DOI:** 10.1186/s13063-021-05912-3

**Published:** 2021-12-20

**Authors:** Brad W. Brazeau, David C. Hodgins, John A. Cunningham, Kylie Bennett, Anthony Bennett

**Affiliations:** 1 Department of Psychology, University of Calgary, Calgary, Canada; 2 National Addiction Centre, Institute of Psychiatry, Psychology, and Neuroscience, Kings College London, London, UK; 3 Centre for Addiction and Mental Health, Toronto, Canada; 4 Department of Psychiatry, University of Toronto, Toronto, Canada; 5 Australian National University, Canberra, Australia; 6 eHub Health Pty Ltd, Goulburn, Australia

**Keywords:** Trial protocol, Self-directed intervention, Brief intervention, Problem gambling, Gambling disorder, Online intervention, Randomized controlled trial, Motivational interviewing

## Abstract

**Background:**

Despite the success of gold standard cognitive-behavioral therapy for problem and disordered gambling, the majority of individuals with gambling problems do not seek or receive professional treatment. Thus, the development of less intrusive self-directed interventions has been encouraged. Bibliotherapy for problem gambling has shown promise, both alone and in combination with motivational interviews, but there is still a lack of online self-directed intervention research. The current randomized controlled trial proposes to assess the additive benefit of a single digital motivational interview delivered in conjunction with an online self-directed treatment program for problem gambling and gambling disorder.

**Methods:**

A two-arm randomized controlled trial will be conducted, wherein eligible participants (*N*=270) will be recruited across Canada via internet advertisements posted to several platforms. All participants will receive access to an online self-directed gambling intervention program. Participants will be randomly assigned to either complete the online program alone or receive a digital motivational interview, conducted through an online audioconferencing platform (i.e., Microsoft Teams) to supplement the online program. The primary outcomes of gambling severity, frequency, and expenditures will be tracked along with secondary outcomes (i.e., depression, anxiety, general distress, alcohol use, and online program user data) over a 24-month period. It is expected that participants in both groups will experience a reduction in symptoms across the board, but more substantial improvements will be observed in the group that receives a supplemental motivational interview.

**Discussion:**

The results of this trial will expand upon prior gambling intervention research by informing best practices for the provision of online self-help for problem gambling.

**Trial registration:**

ISRCTN ISRCTN13009468. Registered on 7 July 2020.

**Supplementary Information:**

The online version contains supplementary material available at 10.1186/s13063-021-05912-3.

## Introduction

### Background and rationale

Most adults worldwide occasionally participate in some form of gambling, and this remains a leisure activity for them. However, some individuals’ gambling involvement escalates, and they continue gambling in an effort to recover their financial losses. Gambling disorder (GD) is diagnosed when persistent and recurrent gambling behaviors, such as chasing losses, lead to significant impairment or distress [1]. There are numerous ramifications following problematic gambling behavior at both individual and societal levels. For example, problem gambling often precedes financial strain, relationship difficulties, and criminal activity [2]. Additionally, those with GD experience a high level of comorbidity with mood, anxiety, personality, and other addictive disorders, as well as suicidality [3].

According to the diagnostic criteria in the *Diagnostic and Statistical Manual of Mental Disorders* (DSM-5), the lifetime prevalence of GD ranges from 0.4 to 1% [1]. In Canada and Alberta, past-year estimates of problem gambling approximate 2.4% and 2.8%, respectively [4]. Despite its high prevalence, as many as 85% of individuals with GD do not seek or receive professional treatment [5] for reasons such as shame, stigma, and a desire to solve the problem independently [6]. Considering the large proportion of those with GD who do not receive treatment, research into means of reaching this population without requiring extensive clinician contact has been encouraged.

Previously conceptualized as an impulse control disorder, GD is now included under non-substance-related addictive disorders in the DSM-5 [1]; thus, treatment regimens for GD are often grounded in models originally designed for substance-related addictions. Consistent with a stepped-care model of treatment for addictive disorders, a multitude of low-intensity interventions have been developed. Such interventions are intended to be minimally intrusive, yet allow for increasing intensity if necessary [7, 8]. For example, if non-assisted recovery fails (i.e., no intervention), treatment users have the option to progress to self-directed interventions (e.g., workbooks), brief interventions (e.g., motivational interviews), or more intensive interventions (e.g., weekly cognitive-behavioral therapies). In sum, this model allows for treatment to be tailored to individual needs without being overly intrusive.

Several self-directed interventions for GD have been established [7–9]. Notably, Hodgins and Makarchuk [10] developed a self-help workbook for gambling problems based on cognitive-behavioral principles. The activities within the workbook are organized into four modules: self-assessment, goal setting, goal implementation, and goal maintenance. Among other activities, workbook users are able to assess the frequency and severity of their gambling behaviors, set reduction- or abstinence-based goals, and plan for future urges, triggers, or potential relapses. This particular workbook has demonstrated efficacy in multiple trials [11–14]. The results of these trials suggest that the efficacy of this workbook is maintained even without any therapist contact (e.g., motivational interviewing), although the effects are not as strong.

In the first trial examining the efficacy of Hodgins and Makarchuk’s workbook [10], Hodgins and colleagues [13, 14] randomized participants to one of three trials: workbook-only, workbook plus telephone motivational interview, or a 4-week waitlist control condition. Follow-up assessments were conducted at 1, 3, 6, 12, and 24 months. Results indicated that both the workbook-only and workbook plus motivational interview groups experienced reductions in gambling frequency and severity compared to the waitlist control group at all time points except the 12-month follow-up. The improved outcomes were more substantial for the workbook plus motivational interview group. Although still significant, group differences were smaller at 6- and 24-month follow-ups compared to 1- and 3-month follow-ups [13, 14]. However, since differential gains were still observed at 24 months, the results suggest that the benefits of self-directed interventions are still apparent in the medium- to long-term, particularly when supplemented with a single session of motivational interviewing. Interestingly, a subsequent trial by Hodgins and colleagues [12] observed no incremental improvement in outcomes when additional motivational interviews were provided. These findings imply that a single motivational interview is enough to enhance the benefits of self-directed workbooks, and additional motivational interviews may not be necessary.

Internet interventions offer a cost-effective alternative to paper workbooks and traditional face-to-face interventions. Guided and unguided internet interventions have been largely successful when used to improve a variety of health conditions, such as diet, physical activity, tobacco use, and excessive alcohol use [15]. However, despite the promising development of self-guided paper workbooks for disordered gambling, both alone and in combination with other brief treatments, there is a lack of research exploring the impact of self-guided internet interventions in this area [16]. Of the research that has been done, much is not optimistic. Paradoxically, the lack of therapist contact, which was originally considered necessary to minimize in self-directed interventions, may be contributing to the deficits in treatment engagement [16]. The challenge that remains is providing sufficient therapist contact to facilitate treatment engagement without deterring a population that has strong preferences to limit such contact.

Previous research has combined self-guided internet interventions with brief therapist contact in the form of telephone instructions [17] or personalized feedback via email [18], but no significant effects were found. However, Carlbring and Smit [19] did find differential improvement when online self-guided treatment was paired with personalized emails and brief weekly phone calls providing instructions and support. Taken together, the literature suggests that therapist contact plays a crucial role in the recovery process, but the active component of contact comes in the form of brief support or the awareness that a professional is monitoring and encouraging their behavior changes.

### Objectives and major research questions

The primary purpose of this study is to expand the research on self-guided internet interventions for disordered gambling and explore whether they can have a more pronounced benefit when paired with minimal supportive clinician contact provided digitally. This contact will come in the form of motivational interviewing, which has been successfully paired with bibliotherapy in multiple prior trials [12–14] but has yet to be paired with online self-directed interventions for problem gambling. The specific objectives are twofold: (1) replicate, in the context of virtual care, the finding that supplemental motivational enhancement improves self-directed gambling treatment outcomes; and (2) test the hypothesis that supplemental motivational enhancement increases online self-help treatment adherence and engagement of individuals seeking treatment for gambling problems.

The current study will randomly assign participants to one of two conditions: internet workbook only (IO) or internet workbook plus motivational interview (IMI). In line with prior gambling intervention trials, primary outcomes will include gambling frequency, problem severity, and expenditures, while secondary outcomes will include measures of mental health, time spent on the self-help site, and participant feedback. The hypotheses for the current trial are:

#### Hypothesis 1 (H1)

Those in both the IO and IMI treatment conditions will experience a reduction in gambling frequency over the course of treatment. However, this reduction is expected to be more pronounced for those in the IMI condition.

#### Hypothesis 2 (H2)

Those in both the IO and IMI conditions will experience a reduction in gambling problem severity over the course of treatment. However, this reduction is expected to be more pronounced for those in the IMI condition.

#### Hypothesis 3 (H3)

Those in both the IO and IMI conditions will experience a reduction in gambling expenditures over the course of treatment. However, this reduction is expected to be more pronounced for those in the IMI condition.

#### Hypothesis 4 (H4)

Reductions in gambling frequency and severity for both the IO and IMI conditions will be positively correlated with time spent using the online self-help tools (i.e., more time spent online will be associated with greater reductions in gambling frequency and severity).

#### Hypothesis 5 (H5)

The IMI group will demonstrate greater adherence (i.e., more modules completed on the self-help website) compared to the IO group.

In addition to the four hypotheses listed above, one exploratory research question will be examined to determine the impact of each treatment condition on participants’ attitudes toward treatment. Attitudes are important to probe considering common pre-existing reluctance to engage in professional treatment in this population. The following research question will be examined:

#### Question 1 (Q1)

Will there be a difference in online workbook ratings between participant intervention groups?

## Methods

### Ethical approval and compensation

This study, including the methods and design, was approved by the University of Calgary Conjoint Faculties Research Ethics Board (CFREB), REB20-0568, in May 2020. Any modifications to the protocol, including changes to the objectives, design, sample size, or study procedures, will be agreed upon by all investigators and submitted for ethical review and approval prior to implementation. As of October 2021, two protocol amendments have been approved and implemented: (a) addition of a 24-month follow-up period to the existing 3-, 6-, and 12-month follow-up plan; and (b) requirement of MI session completion prior to, rather than following, the first CAD $10 compensation.

Participants will be financially compensated an electronic gift card valued at CAD $10 following: (1) confirmation of eligibility; (2) completion of the baseline assessment; (3) creation of an account with the online self-help program; and (4) completion of a motivational interview (if assigned to that group). Participants will also be remunerated with an electronic gift card valued at CAD $30 after each of four follow-up assessments have been completed in full; in total, participants could receive CAD $130 in electronic gift cards. Participants will have the ability to choose electronic gift card(s) from a number of local stores and restaurants. There is a possibility that some participants will sell their gift cards to finance gambling or otherwise take advantage of the compensation offered. We will mitigate this by screening for duplicate participants and using electronic (versus physical) gift cards.

### Confidentiality

Anonymity cannot be guaranteed, due to the use of email addresses (for online accounts and e-gift card compensation) and names to match interview data with online data. At the outset, participant data will be linked via personal information provided. Data from the online workbook will be linked with personal and survey data via unique participant identification numbers. Participants will be informed of these limits to anonymity during the consent process. After all data has been linked and participants have been compensated, personally identifying information (in a master spreadsheet) will be permanently deleted, with the exception of age and sex. This will leave only the anonymized data in an SPSS file. Participant data will be kept completely confidential unless there is (a) acute risk of harm to self or others (e.g., suicidal plans) or (b) court-ordered subpoena/other legal demands for data. If there is a high risk to self or others disclosed during an MI session, participants will be informed that members of the research team may contact local authorities or medical services to prevent harm.

### Participant recruitment and randomization

Participants will be recruited via targeted internet advertisements (i.e., “Are you concerned about your gambling? Study includes free and confidential online help for your gambling”). These advertisements will be distributed online (e.g., Facebook; Kijiji; YouTube; Google; Twitter; Reddit) to media users across Canada. To meet inclusion criteria, participants must (a) be a Canadian resident; (b) be 18 years of age or older; (c) have gambled at least once within the last month; (d) score 5 or more on the Problem Gambling Severity Index (PGSI) [20]; and (e) not currently be involved in treatment for their gambling. As with most prior gambling intervention trials, use of psychiatric medication will not be an exclusion criterion, but will not be assessed or monitored in this study. Prospective participants will be excluded if they are unable or unwilling to (a) provide their phone number and email address or (b) access the online program to create an account.

Individuals who are interested will be automatically redirected from a social media advertisement to an online eligibility screening questionnaire on Qualtrics. Those who are found to be ineligible will be thanked for their interest and directed to alternative resources for gambling help without any contact from the research team. Those who meet eligibility criteria will be automatically prompted to provide consent and contact information within Qualtrics, and then complete the baseline assessment. Qualtrics will be monitored daily for completed surveys that meet eligibility criteria and indicate consent. Eligible participants will be randomized by the research team to one of the two treatment groups and notified of their group assignment via email. Those selected to receive a motivational interview will also be invited to schedule their session. As previously mentioned, participants will not receive the initial compensation until the aforementioned steps have been completed. Those who complete at least one but not all of these steps will be sent a maximum of two reminder emails. Eligible participants can choose to be informed of the trial results via email upon its completion. Regardless, any publications and presentations resulting from this trial will be agreed upon by all investigators.

#### Participant validation

Email and IP addresses associated with survey responses will be checked for duplicates to ensure each participant only completes each survey once. Additionally, a VPN block and reCAPTCHA system will be implemented within each survey to prevent the enrolment of ineligible and fake participants, respectively. Finally, a randomly selected PGSI question will be presented at the end of the eligibility questionnaire; only participants whose response to this question matches the corresponding question in the initial set of PGSI questions will be enrolled.

Recruitment and retention of individuals seeking treatment for gambling problems can be a challenge. For example, a recent trial that assessed a brief versus extended self-directed online intervention found that only 66% of participants overall completed all three follow-up assessments. Furthermore, over 40% in the extended intervention never even accessed the self-help website [21]. To ensure an adequate number of participants are recruited and retained, this trial proposes to employ (a) nationwide recruitment; (b) compensation after the baseline assessment and each follow-up; and (c) multiple points of contact via email between participants and researchers. Additionally, engagement tends to be higher for briefer self-directed interventions (such as the current one) compared to extended self-directed interventions or professional treatment [21, 22]. Follow-up rates also appear to be higher when contact information beyond email addresses (e.g., phone numbers) are collected, as the least committed treatment seekers likely do not wish to provide this information [23]. Finally, the collection of participant feedback via the IEUQ survey can serve to guide the design of future online interventions for problem gambling such that attractiveness and uptake are maximized.

This protocol has been developed in accordance with the Standard Protocol Items: Recommendations for Interventional Trials (SPIRIT) statement. A randomized two-arm clinical trial will be conducted (see Table 1; Additional file 1). Participants will be automatically randomized in a 1:1 ratio to one of two groups: an internet only (IO) control group or an internet plus motivational interview (IMI) group. Group randomization will employ computer-generated minimization using the program MINIM [24] and stratified by sex (male; female; other), gambling severity (low-moderate; high), and whether they have been previously treated for their gambling (yes; no) based on stratification strategies used in prior trials [12–14]. Gambling severity, for the purpose of randomization, will be defined based on PGSI scores of low-moderate (score of less than 8) or severe (score of 8 or greater). MINIM will generate the allocation sequence, which it conceals until the moment of assignment (i.e., participant stratification data is entered and MINIM returns the group assignment for each participant individually at the time of assignment).

**Table 1:**
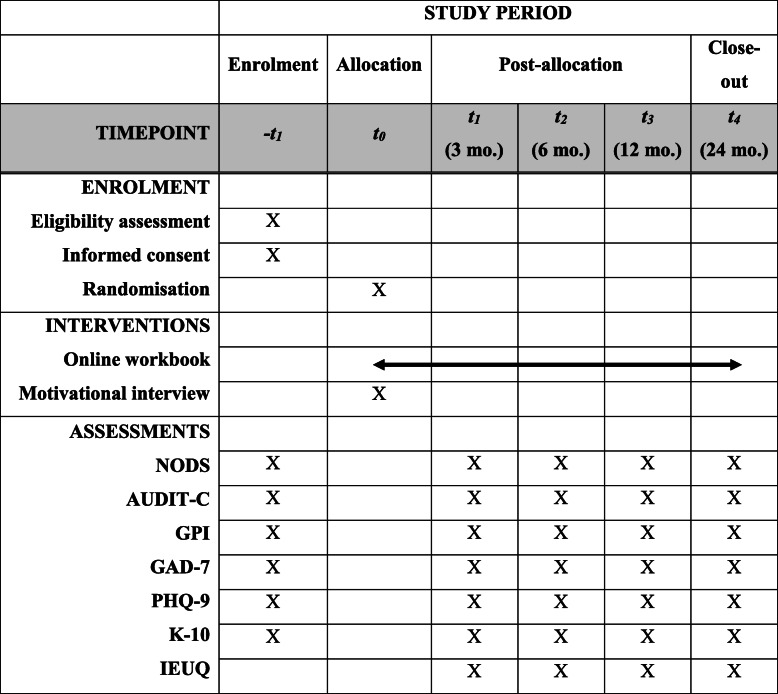
Schedule of enrolment, interventions, and assessments

Participants will be invited via email to complete follow-up assessments at 3, 6, 12, and 24months after their enrolment in the study. They will not be dropped from the study unless they explicitly request to withdraw by contacting the research team as indicated in the consent form. Participants who drop out or require ancillary or post-trial care will be provided with local resources (e.g., contact information for crisis lines, emergency departments, family physicians, etc.) and encouraged to create a non-research account with the online program.

### Intervention conditions

#### Internet only (IO)

This treatment group will be provided with access to an online workbook (www.gamblingselfhelp.com) based on a paper version mentioned earlier [10] that has demonstrated efficacy in multiple trials [11–14]. It consists of cognitive-behavioral self-help tools to reduce problem gambling. These tools are organized into four modules: self-assessment, goal setting, goal implementation, and goal maintenance. The design of the modules allows for the provision of concise cognitive-behavioral strategies for controlling or abstaining from gambling. The activities within each module may be completed in any order and as many times as desired. None of the activities is mandatory. Participants will have unrestricted access to the site for the entire duration of the study in order to mimic other web resources as much as possible in terms of accessibility.

#### Internet plus motivational interview (IMI)

This treatment group will be provided with the same access to the online workbook as those in the IO group, plus one brief (i.e., 30- to 60-min) motivational interview delivered digitally through an online audioconferencing platform (i.e., Microsoft Teams) within the first two weeks of study enrolment. In the event that participants have difficulties with Microsoft Teams, interviews will be conducted via telephone. Participants who refuse the motivational interview will not be compensated with the initial gift card, but will be retained in this group and followed up with in accordance with an intention-to-treat approach (ITT). Motivational interviewing involves the assessment of a client’s readiness for change, followed by the facilitation of behavioral change by exploring ambivalence, building commitment, and eliciting reasons for change [25]. Clinical psychology graduate students will be trained to conduct the motivational interviews and will be compensated with clinical credit hours. All calls will be digitally recorded. Twenty percent of the calls will be assessed for treatment fidelity using a treatment adherence checklist, and they will be rated by two independent raters for reliability. The adherence checklist covers a number of elements that are essential to include (e.g., asking for commitment) and not include (e.g., providing unsolicited advice) during the interview sessions.

### Eligibility assessment

#### Demographics

A variety of demographic questions used in previous gambling trial studies will be asked to screen for eligibility and gather descriptive information in regards to age, sex, education level, income, occupation, ethnicity, marital status, and types of gambling they engage in. They will also be asked whether or not they are currently receiving treatment for their gambling and if they have in the past.

#### Gambling severity

The PGSI [20] will be used to screen for gambling problems prior to study commencement. The PGSI is a 9-item scale used to assess problem gambling severity. Respondents answer a series of questions related to their gambling on a 4-point scale ranging from “never” to “almost always,” and total scores range from 0 to 27. While many prior trials have used a PGSI score of 3 as a cutoff, Currie, Hodgins, and Casey [26] determined that a cutoff score of 5 better differentiates low- from moderate-risk behavior in terms of gambling expenditures. PGSI scores have been shown to correlate highly with other measures of gambling severity (*r* = .83), such as the National Opinion Research Center DSM-IV Screen for Gambling Problems (NODS). Additionally, the PGSI has demonstrated good internal consistency (*α* = .84) [20].

### Baseline and follow-up assessments

#### Primary outcomes

##### Gambling frequency and expenditures

Prospective participants will also be asked to estimate the average number of hours they have gambled per month and per gambling session over the last three months, as well as the average amount of money they won or lost per month and per gambling session. These 3-month retrospective self-report questions were adapted from the Gambling Participation Instrument (GPI) [27].

##### Gambling severity

In addition to gambling frequency and expenditures, gambling severity will also be assessed. The NODS [28] is a 17-item measure that uses DSM-IV criteria to assess gambling problems. The 3-month version of the NODS has been previously validated as an outcome measure for gambling intervention research, demonstrating good internal consistency (*α* = .87) [29]. It also correlates highly with other measures of gambling severity (*r* = .86) and moderately with number of days gambled and number of dollars spent (*r* = .50) [30].

#### Secondary outcomes

##### Depression symptoms

The Patient Health Questionnaire-9 (PHQ-9) [31] is a 9-item scale that measures symptoms of depression over the past two weeks. Item response options range from 0 (not at all) to 3 (nearly every day), yielding total scores that range from 0 to 27. The PHQ-9 has shown good internal consistency (*α* = .89) [31]. Note that participants who endorse question 9 on the PHQ-9 (i.e., thoughts that they would be better off dead or hurting themselves in some way) will be directed to an automated response at the end of the survey; this response will encourage these participants to consult a resource (e.g., family physician) or contact a crisis helpline via phone numbers provided to them. They will be explicitly informed that contact from the research team will not follow the automated response.

#### Anxiety symptoms

The Generalized Anxiety Scale-7 (GAD-7) [32] is a 7-item scale that measures symptoms of anxiety over the past two weeks. Each item is responded to using a 4-point Likert scale, and total GAD-7 scores range from 0 to 21. The GAD-7 has demonstrated excellent internal consistency (*α* = .92) [32].

#### General psychological distress

The Kessler Psychological Distress Scale-10 (K-10) [33] is a 10-item scale used to assess nonspecific psychological distress-related symptoms over the past four weeks. Response options span a 4-point scale ranging from “none of the time” to “all of the time,” and total scores range from 0 to 40. The K-10 has demonstrated high internal consistency (*α* = .78) [33].

#### Alcohol consumption

The Alcohol Use Disorder Identification Test – Consumption (AUDIT-C) [34] is a 3-item short-form of the full AUDIT that only measures alcohol consumption. Using a cut-off score of 3, the AUDIT-C has sensitivity of 98% and specificity of 57% for identifying active alcohol abuse or dependence [34].

#### Program evaluation

The Internet Evaluation and Utility Questionnaire (IEUQ) [35] is a 15-item scale used to measure participants’ experiences and perceptions of the online self-directed workbook. Questions include those related to ease of use, convenience, engagement, mode of delivery, and likelihood of returning. The IEUQ has demonstrated adequate internal consistency (*α* = .69) [35].

#### User data

In addition to the measures described above, user data will also be collected from the workbook website. These data will allow us to examine how much time participants spend on the site and individual modules, as well as which modules are in progress or completed, and the number of times participants logged on to access the online workbook.

#### Additional help-seeking

Upon completion of the treatment, participants will be asked to indicate whether or not they will seek further treatment or support for their gambling problems. They will also be asked to indicate what type, if any, that they intend on seeking. Options will include engagement in face-to-face therapy, attendance of support groups, and speaking to family or friends.

### Blinding

Participants will be informed during the consent process that they will be randomly assigned to one of the two intervention conditions. Neither intervention condition will be described as superior. Baseline assessment will occur prior to randomization and follow-up assessments will occur after. Motivational interview sessions will be conducted after randomization, so both participants and interviewers will be aware of participant assignment.

### Data monitoring

A data monitoring committee (DMC) is not necessary for this trial because it (a) poses minimal risk of research-related harm; (b) is unblinded; (c) involves a single data collection site; and (d) does not involve conflicts of interest. The research team, under the guidance of the CFREB, have the responsibility to monitor safety, efficacy, and validity throughout the conduct of the study. The trial will not be audited.

### Sample size estimation

The sample size was determined based on previous work exploring self-directed gambling interventions [11–14] and was computed using G*Power [36] on the basis of H1–H3. The estimation method was based on a repeated-measures between-factors ANOVA, which serves as a simpler derivative of the general linear model that the primary analyses will be based on [37, 38]. Two superiority hypotheses using three primary outcome measures will be analyzed using conventional two-tailed tests with thresholds of power = .80 and *α* = .05. Bonferroni corrections will be calculated to account for multiple comparisons. Correlations of *r* = .50 between baseline and follow-up data were accounted for [16]. A sample size of 108 participants per group will permit statistical detection of small effect sizes (Cohen’s *d* = 0.20 for continuous measures) at the specified significance level. This translates to differential detection of approximately 2 days less gambling per month and a 1-point decrease in NODS scores [37]. After accounting for 20% attrition over 12 months, the resulting planned sample size is 270 participants.

### Data analyses

All statistical analyses will be conducted using SPSS version 26.0 at 12- and 24-month follow-up time points. Following completion of the 24-month follow-ups, the trial will be terminated. If baseline data are missing completely at random, they will be handled using the full imputation maximum likelihood approach to estimate means, variances, and covariances. Missing data at follow-ups will be handled with the multiple imputation method. Analyses will be conducted using the intention-to-treat (ITT) approach. Bivariate comparisons of all demographics and outcome measures will be conducted to explore differences between groups at baseline. Chi-square tests of independence will be used to compare groups on categorical measures, while *t* tests will be used to compare groups on continuous measures. Any primary outcome variables with significant differences between groups will be controlled for by entering them in as covariates in primary and secondary analyses. It is likely that the primary outcome variables will appear non-normally distributed when assessed with q-q plots and Shapiro-Wilk tests; in these cases, data will be appropriately transformed to achieve normality. If the data transformations are unsuccessful, separate nonparametric analyses will be conducted as appropriate instead of the respective planned analyses outlined below. Additionally, chi-square tests of independence will be used to determine if attrition rates differ in terms of treatment group or baseline characteristics.

H1 and H2 will be analyzed using generalized equation estimation (GEE) to determine if there are group (IO; IMI) differences in change over time. Outcome variables will include gambling frequency (i.e., number of days gambled in the last month), expenditures (i.e., average number of dollars lost per gambling day), and severity (i.e., NODS score). GEE is advantageous because it can account for the natural correlations over time in longitudinal data [38].

To aid in clinical significance and translation, H1 and H2 will also be analyzed categorically with two separate logistic regressions. Both analyses will include Group and Time as binary predictors. The analysis to test H1 will include gambling disorder status (meets criteria; does not meet criteria) as the binary outcome. The analysis to test H2 will include recovery status (abstinence; improvement; no improvement) as the multinomial outcome. Improvement is defined as at least a 50% reduction in average number of dollars lost per gambling session.

H3 will be analyzed with a bivariate Pearson correlation between time spent on the site and change in dollars spent per gambling day from baseline to follow-ups.

H4 will be analyzed with two bivariate comparisons. Two unpaired *t* tests will be conducted to determine if the adherence rates differ by group after the 12- and 24-month follow-ups. Adherence will be measured in two ways: (1) number of modules completed on the program and (2) total time spent on the program website.

To test the exploratory research question, bivariate comparisons will be conducted. Q1 will be analyzed with an unpaired *t* test comparing group treatment ratings at each of the follow-up periods (3, 6, 12, and 24 months).

## Discussion

### Strengths and limitations

Given that most individuals with gambling problems do not seek professional treatment, this intervention’s basis in a stepped-care model is a major strength. In addition to its low intensity, this intervention offers the potential for cost-effectiveness, user-friendliness, and widespread accessibility. Finally, another strength is that gambling severity will be analyzed both continuously and categorically. The categorical analysis classifies participants’ NODS scores (i.e., meets criteria for GD; does not meet criteria for GD) and changes in gambling expenditures (i.e., no improvement; improvement; abstinence). This strategy effectively aids in clinical significance and practical translation of findings by translating raw scores to real-world taxonomies, thereby simplifying the implications of the results.

This study presents with some limitations as well. Of particular importance is the fact that the online program modules can be completed in any order, and not all of the activities must be completed. This program feature does not permit analysis of the degree to which the benefits are conditional upon the order that activities or modules are completed. While user data will still be collected, and some participants may complete the modules in order, there is no way to standardize the order of completion at this time. However, this feature may be viewed as beneficial, since the paper and pencil version of this workbook discussed earlier [10–14] could also be completed in any order; it thereby allows for a more accurate comparison of efficacy between the paperback and online versions. Such comparisons, although imprecise, are important given the absence of a true control group to directly assess program efficacy in this study.

Another limitation is the use of a simplified MI adherence checklist. Best practices recommend much more thorough coding schemes, such as the Motivational Interviewing Treatment Integrity (MITI) protocol. One argument in support of the briefer checklist is that it permits efficient coding of entire interviews. In contrast, more sophisticated integrity protocols are designed to comprehensively code only portions of interviews. Since therapist adherence can fluctuate throughout a single MI session [39, 40], the brief checklist allows for more efficient and representative coding of entire interviews.

## Trial status

Protocol version: 3 (19 October 2021).

Date recruitment began: 19 August 2020.

Approximate date recruitment will be completed: 31 March 2022.

## Supplementary Information

**Additional file 1.** Recommended items to address in a clinical trial protocol and related documents.

## Data Availability

There are no applicable data since this is a protocol paper. The study materials are available from the corresponding author upon request. The final data resulting from the trial will be accessible to all investigators and available upon request from the corresponding author.

## Abbreviations

AUDIT-C: Alcohol Use Disorders Identification Test – Consumption
DSM-5: Diagnostic and Statistical Manual of Mental Disorders, 5^th^ Edition
GAD-7: Generalized Anxiety Disorder 7-item scale
GPI: Gambling Participation Instrument
IEUQ: Internet Effectiveness and Utility Questionnaire
IMI: Internet plus motivational interview condition
IO: Internet-only condition
K-10: Kessler Psychological Distress Scale
NODS: National Opinion Research Centre DSM-IV Screen for Gambling Problems
PGSI: Problem Gambling Severity Index
PHQ-9: Patient Health Questionnaire 9-item scale
RCT: Randomized controlled trial
SPIRIT: Standard Protocol Items: Recommendations for Interventional Trials
